# Recurrent Myalgia, Dark Urine, and Exercise Intolerance: Glycogen Storage Disease Type X Diagnosed Through Gene Sequencing Panel

**DOI:** 10.7759/cureus.70175

**Published:** 2024-09-25

**Authors:** Yutaka Furuta, John A Phillips

**Affiliations:** 1 Medical Genetics, Vanderbilt University Medical Center, Nashville, USA

**Keywords:** dna sequencing-based gene panel testing, exercise intolerance, glycogen storage diseases x, metabolic myopathies, recurrent myalgia

## Abstract

Evaluation of a single episode of exercise-induced myalgia, dark urine, and elevated creatine phosphokinase (CPK) levels relies primarily on clinical history and physical examinations. However, recurrent episodes necessitate further investigations for potential genetic conditions. This is a case of an 11-year-old male who presents with recurrent myalgia, dark discolored urine, and exercise intolerance for the past year. The initial examination revealed hematuria and a mild elevation of transaminases. Referral to nephrology showed elevated CPK level, prompting further evaluation by neurology. A DNA sequencing-based neuromuscular genetic panel identified a heterozygous pathogenic variant and two variants of uncertain significance in his *PGAM2* genes, leading to a diagnosis of glycogen storage disease X. Metabolic myopathies should be considered in children with recurrent exercise-induced myalgia and dark discoloration of urine. Screening for myoglobinuria, elevated plasma CPK levels, and characteristic acylcarnitine profiles should be considered when clinically indicated. DNA sequencing-based gene panel testing can serve as a non-invasive alternative to muscle biopsy for diagnosing metabolic myopathies when suspicion is high.

## Introduction

Myalgia, or muscle pain, is a common complaint in both children and adults and could be associated with additional features such as exercise-induced myalgia and dark urine. Creatine phosphokinase (CPK) is a useful marker in patients with myalgia. It is an enzyme that is primarily present in muscles and becomes elevated when muscle tissue is injured or stressed. Severe muscle breakdown, due to various causes, can lead to rhabdomyolysis, a life-threatening condition characterized by muscle necrosis, myoglobinuria, acute renal failure, electrocyte derangement, and arrhythmia [1]. Evaluation of a single episode of exercise-induced myalgia, dark urine, and elevated CPK level typically relies on clinical history and physical examinations [1]. In children, common causes of elevated CPK include infections, vigorous exercise, medications, and toxins [2]. However, recurrent episodes warrant further investigations to consider potential underlying genetic conditions, particularly inherited metabolic myopathy, including neuromuscular diseases and inborn errors of metabolism [3,4]. Neuromuscular disorders, such as muscular dystrophy, inflammatory myopathies, and malignant hyperthermia susceptibility, can lead to myopathy. Additionally, glycogen storage diseases (GSD), fatty acid oxidation disorders (FAOD), and mitochondrial disorders are inborn errors of metabolism that can also cause myopathy. 

In recent years, advances in technologies such as next-generation sequencing (NGS) have led to the identification of numerous novel causative genes for various diseases, enabling definitive diagnosis for conditions that were previously undiagnosable [5]. NGS encompasses several approaches, including deoxyribonucleic acid (DNA) sequencing-based gene panel testing, exome sequencing, and genome sequencing. For individuals presenting with specific clinical phenotypes, such as myopathy, DNA sequencing-based gene panel testing is often the preferred diagnostic tool due to its focused and targeted approach.

We report here a pediatric case of recurrent myalgia, dark discolored urine, and exercise intolerance caused by glycogen storage disease type X (GSD X), diagnosed through DNA sequencing-based gene panel testing. GSD X is a rare form of glycogen storage disease, and its diagnosis requires molecular genetics testing. This case highlights the impact of DNA sequencing-based gene panel testing in detecting rare metabolic myopathies in individuals presenting with such recurrent episodes.

## Case presentation

An 11-year-old boy presented to his primary pediatrician with a history of leg cramps, dark discolored urine, and tiring easily during exercise. These symptoms began approximately a year ago, especially with basketball, baseball, and even 10-second sprints. In the general pediatric clinic, he was found to have hematuria (3+ blood) on urinalysis and mild elevation of transaminases. His complete blood counts, electrolytes, glucose level, and renal function were normal. Given the presence of hematuria, he was referred to pediatric nephrology for further evaluation of potential renal disease. Apart from exercise, no known triggers were identified, including recent illness, injury, new medications, or surgery. He also denied fever, pain during voiding, muscle weakness, tingling, or numbness. He does not take any prescribed medications or supplements. His home, education (ie, school), activities/employment, drugs, suicidality, and sex (HEADSS) exam was negative for recreational drug and alcohol use. No biological family history was available because he was adopted in infancy. Physical examinations were unremarkable without hepatomegaly or muscle tenderness. The evaluation revealed an elevated creatinine level of 1.01 mg/dL (reference range: 0.52-0.80 mg/dL) and a CPK level of 738 units/L (reference range: 29-168 units/L). His electrolytes, glucose level, and hepatic function were unremarkable. Urinalysis was negative for blood. Renal ultrasonography revealed no nephrolithiasis. Due to concerns about a neuromuscular disorder based on his elevated CPK level, he was referred to pediatric neurology for further evaluation. He still endorsed his myalgia, fatigue, and visible dark urine recur with even brief vigorous exercise. His vital signs were within normal range. Physical examination showed that he was well-nourished and well-developed. No dysmorphic features were appreciated. Cardiac examination revealed normal heart sounds, with no murmurs, rubs, or gallops. His respirations were unlabored, and his lungs were clear to auscultation bilaterally. His abdomen was soft and flat, with no hepatomegaly or splenomegaly. Costovertebral angle tenderness was absent. Neurologic examinations and muscle strength were intact. Given his recurrent episodes of exercise-induced myalgia, dark-colored urine, and elevated CPK levels, a genetic neuromuscular disorder, particularly inherited metabolic myopathy, was suspected. A DNA sequencing-based neuromuscular genetic panel was obtained, which showed a heterozygous pathogenic variant (p.Try78*) and two variants of uncertain significance (p.Arg162Pro, p.Glu128Lys) in his *PGAM2* genes.

## Discussion

Differential diagnosis

His presentations of recurrent exercise-induced myalgia, fatigue, dark coloration of urine, and elevated CPK levels raise suspicion for inherited metabolic myopathy. Common causes of metabolic myopathies in children include GSD and FAOD (Table 1) [3]. Muscular type GSDs (e.g., type V, VII, X, phosphoglycerate kinase 1 deficiency) are characterized by exercise intolerance with myalgia and myoglobinuria [4]. GSD V (McArdle disease) is characterized by a “second-wind” phenomenon, which is an improvement of myalgia and fatigue after a few minutes as muscle metabolism transitions from using carbohydrates as a source of energy to using fatty acids [6]. GSD VII (Tarui disease) can be associated with hemolytic anemia [7]. GSD X is very rare and presents similar to GSD V [8]. Most GSDs are inherited by autosomal recessive mode. In contrast to muscular-type GSDs, patients with FAOD can tolerate short-duration and intense exercise (Table 2) [9]. In this case, his symptoms were precipitated by short-duration burst exercises, which make FAOD less likely. Another differential diagnosis is neuromuscular disorders, including muscular dystrophy, inflammatory myopathies, and malignant-hyperthermia susceptibility (e.g., RYR1 mutation) [1]. Muscular dystrophies are a diverse group of conditions resulting in progressive muscle atrophy and weakness with elevation of CPK level [10]. These conditions can also cause exertional myalgia and myoglobinuria. 

**Table 1:** Common causes of creatine phosphokinase (CPK) in children In children, common causes of elevated CPK include infections, muscle strains, vigorous exercise, traumatic injuries, environmental exposures, medications, and toxins. Genetic conditions, such as neuromuscular diseases and inborn errors of metabolism, can also contribute to elevated CPK levels.

	Category	Example
Common causes	Infections	Viral infection
	Muscle strain, excessive activity, trauma, environmental exposure	Vigorous exercise, injury, hyper/hypothermia
	Medication, toxin	Statin
Inherited causes (rare)	Inherited neuromuscular disorders	Muscular dystrophy, inflammatory myopathies, and malignant-hyperthermia susceptibility (eg., RYR1 mutation)
	Inborn errors of metabolism	Glycogen storage diseases (GSD), fatty acid oxidation disorders (FAODs), mitochondrial disorders

**Table 2:** Comparison of myopathic chrematistics between fatty acid oxidation disorders (FAODs) and muscular-type glycogen storage diseases (GSD) Most FAODs and GSDs are inherited in an autosomal recessive pattern. In contrast to muscular-type GSDs, patients with FAODs can tolerate short-duration, intense exercise. The second-wind phenomenon is commonly seen in muscular-type GSDs but not in FAODs. While CPK levels in FAODs are typically normal between symptomatic episodes, they can remain elevated between episodes in muscular-type GSDs.

	FAOD	Muscular type GSD
Inheritance pattern	Autosomal recessive	Autosomal recessive, X-linked (type IX)
Duration of exercise needed to trigger symptoms	Longer (> 30min)	Shorter (< 30 min)
Second-wind phenomenon	No	Often present
Creatine phosphokinase (CPK) levels	Normal between episodes of symptoms	Elevated even between episodes of symptoms

Diagnosis

He was referred to our genetics clinic for further evaluation. His pathogenic variant and the two variants of uncertain significance were thought to be on opposite alleles. Given his clinical correlation, one or both of the variants of uncertain significance are now considered likely pathogenic and are believed to result in GSD X. Of note, his biological parental genetic testing was unavailable.

GSD X (OMIM #261670) is a very rare type of GSD that is associated with the *PGAM2* gene, encoding phosphoglycerate mutase [8]. This enzyme is essential for the conversion of 3-phosphoglycerate to 2-phosphoglycerate during glycolysis. This disorder is characterized by an autosomal recessive mode of inheritance. Common manifestations include myoglobinuria, muscle cramps, exercise intolerance, myalgia, and rhabdomyolysis with elevation of CPK level. Symptoms are usually induced only by strenuous exercise, particularly bursts of vigorous exercise such as splints [11,12]. A common onset is in childhood or teenage years [8]. GSD X presents with clinical features similar to those of GSD V, making it difficult to differentiate between the two clinically [8]. Therefore, confirmation through molecular analysis is essential for an accurate diagnosis [11]. Due to the rarity of the condition and the limited number of patients, the prognosis remains unclear. The mainstay of management of GSD X is hydration and avoidance of strenuous exercise [11]. In addition to supportive care, there is currently no definitive treatment for GSD X, and further research is both expected and necessary. 

Glycogen is an important energy source for skeletal muscle during exercise. In individuals with muscular-type GSDs, they are not able to break down glycogen, which results in exercise intolerance, muscle cramps, and even rhabdomyolysis [9]. In the initial phase of muscle contractions, particularly isometric contractions due to vigorous exercise (e.g., weightlifting, sprinting), glycogen is the primary substrate used for energy production [9]. For this reason, these symptoms are induced within minutes of hard exercise (Table 2). However, when physical activity is sustained, metabolism switches to the consumption of fatty acids to spare essential glucose requirements of the brain.

To evaluate a diagnosis of patients with recurrent exercise-induced myalgia and dark-colored urine, a case-based, tailored approach should be considered [1]. Tests to consider include plasma acylcarnitine profiles, CPK levels, and urine organic acids. When clinically necessary, molecular analysis and, occasionally, muscle biopsy and enzyme assay could prove beneficial [13]. In this case, his diagnosis was confirmed as GSD X through DNA sequencing-based gene panel testing. Non-invasive biochemical studies are useful screening tests, but molecular genetic testing is often required in many cases. While biochemical studies and molecular genetic testing are noninvasive, muscle biopsy and enzyme assays are invasive. In addition, muscle biopsy is helpful in evaluating neuromuscular disorders. In this case, a non-invasive DNA sequencing-based gene panel test was done rather than a muscle biopsy to diagnose the metabolic myopathy that was suspected. 

Clinical pearls

Consider metabolic myopathies in pediatric patients with recurrent episodes of exercise-induced myalgia and dark-colored urine.

Screening tests for myoglobinuria, elevated plasma CPK levels, and characteristic acylcarnitine profiles should be considered if clinically indicated. 

DNA sequencing-based gene panel testing can be an alternative non-invasive diagnostic test instead of muscle biopsy when there is a strong suspicion of metabolic myopathies.

## Conclusions

We report a case of an 11-year-old male with recurrent myalgia, dark discolored urine, and exercise intolerance who was diagnosed with glycogen storage disease type X (GSD X) through DNA sequencing-based gene panel testing. This accurate diagnosis resulted in a referral to genetics and appropriate management, including hydration, avoidance of strenuous exercise, and genetic counseling. This case highlighted that metabolic myopathies should be considered in pediatric patients with recurrent exercise-induced myalgia and dark discoloration of urine. Screening tests for myoglobinuria, elevated plasma CPK levels, and characteristic acylcarnitine profiles should be considered if clinically indicated. DNA sequencing-based gene panel testing can be an alternative non-invasive diagnostic test instead of muscle biopsy in the setting of high suspicions for metabolic myopathies.
